# Zinc-Solubilizing *Streptomyces* spp. as Bioinoculants for Promoting the Growth of Soybean (*Glycine max* (L.) Merrill)

**DOI:** 10.4014/jmb.2206.06058

**Published:** 2022-10-17

**Authors:** Chanwit Suriyachadkun, Orawan Chunhachart, Moltira Srithaworn, Rungnapa Tangchitcharoenkhul, Janpen Tangjitjareonkun

**Affiliations:** 1 Thailand Bioresource Research Center, National Center for Genetic Engineering and Biotechnology (BIOTEC), National Science and Technology Development Agency (NSTDA), Pathum Thani, 12120, Thailand; 2 Division of Microbiology, Department of Science, Faculty of Liberal Arts and Science, Kasetsart University Kamphaeng Saen Campus, Nakorn Pathom, 73140, Thailand; 3 Graduate School, Suan Dusit University, Bangkok 10300, Thailand; 4 Department of Resources and Environment, Faculty of Science at Sriracha, Kasetsart University, Sriracha Campus, Chonburi, 20230, Thailand

**Keywords:** Zinc-solubilizing rhizobacteria, Streptomyces, bioinoculants, soybean

## Abstract

Zinc-solubilizing bacteria can convert the insoluble form of zinc into soluble forms available to plants. This study was conducted to isolate and screen zinc-solubilizing actinobacteria from rhizosphere soils and to assess their effect on vegetable soybean growth. In total, 200 actinobacteria strains belonging to 10 genera were isolated from rhizosphere soil samples. Among these isolates, four showed zinc solubilization with solubilizing index values ranging from 3.11 to 3.78 on Bunt and Rovira agar supplemented with 0.1% zinc oxide. For the quantitative assay, in broth culture, strains CME34 and EX51 solubilized maximum available zinc contents of 529.71 and 243.58 μg/ml. Furthermore, indole-3-acetic acid (IAA) and ammonia were produced by these two strains, the strain CME34 produced the highest amount of IAA 4.62 μg/ml and the strain EX51 produced the highest amount of ammonia 361.04 μg/ml. In addition, the phosphate-solubilizing abilities in Pikovskaya’s medium of CME34 and EX51 were 64.67 and 115.67 μg/ml. Based on morphological and biochemical characterization and 16S rDNA sequencing, the strains CME34 and EX51 were closely related to the genus *Streptomyces*. In a greenhouse experiment, single-strain inoculation of *Streptomyces* sp. CME34 or EX51 significantly increased the shoot length, root length, plant dry weight, number of pods per plant and number of seeds per plant of vegetable soybean plants compared to the uninoculated control. These findings facilitated the conclusion that the two *Streptomyces* strains have potential as zinc solubilizers and can be suggested as bioinoculants to promote the growth and yield of soybean.

## Introduction

Tuber crops and legumes, especially cassava, sweet potato, soybean and groundnut, are considered important cash crops and food crops in Thailand. In 2021, the Office of Agricultural Economics (OAE) reported that the production volumes of cassava, soybean and groundnut in Thailand were approximately 35 million tons, 50 thousand tons and 35 thousand tons, respectively [1]. In Thailand, calcareous soils are widely used for cultivating economic crops and zinc deficiency frequently appears in the soil [2-4]. Calcareous soils contain high levels of calcium carbonate that affect low-bioavailable zinc concentrations due to some of the zinc adsorbed on calcium carbonate in this soil [5]. Zinc-deficient soils may reduce crop yields and nutritional quality.

Zinc is a micronutrient required as a structural component or regulatory cofactor of several enzymes and proteins [6]. It is involved in the metabolism of carbohydrates, the synthesis of proteins and chlorophyll, the maintenance of plasma membrane integrity, and the biosynthesis of plant growth hormones, such as auxin [7]. Zinc deficiency in plants causes approximately 50% decreased indole-3-acetic acid (IAA) levels in the shoot apex and young leaves [8]. The symptoms of zinc deficiency in plants include interveinal chlorosis of young leaves, spotting of older leaves, reduced leaf size, and stunted growth [9]. Generally, zinc fertilizers are used to increase the level of zinc fortification in plants. Zinc sulfate (ZnSO_4_) has been widely used as an inorganic fertilizer for soil application because of its high solubility and low cost [10]. However, ZnSO_4_ has a low efficiency of available zinc uptake by plants because the soluble zinc is easily converted into different unavailable forms in soils with relatively poor soil characteristics such as high pH values, calcium carbonate contents or phosphate contents [11]. An alternative solution to this problem is to enhance the availability of zinc using biological agents such as zinc-solubilizing rhizobacteria.

Zinc-solubilizing rhizobacteria are beneficial, root-associated bacteria that can convert unavailable forms of zinc into plant-accessible forms through several mechanisms, with organic acid production such as the production of 2-ketogluconic acid and gluconic acid being a major mechanism [12]. Different zinc-solubilizing rhizobacteria genera have been reported, such as *Bacillus* sp., *Pseudomonas* sp,. and *Gluconacetobacter* sp. [12-15]. Among the various bacterial groups, actinobacteria are a group of gram-positive filamentous bacteria and are a major group in rhizosphere soil populations. Actinobacteria play an important role in promoting plant growth through the production of plant hormones, fixation of nitrogen, and the production of enzymes and bioactive compounds [16, 17]. These bacteria have also been reported to solubilize various insoluble mineral elements into soluble forms available for plant uptake. Among them, the genus *Streptomyces* is considered a predominant producer used in plant growth promoters and biocontrol agents [18]. Earlier studies on actinobacteria genera, such as *Arthrobacter*, *Cellulosimicrobium*, *Kocuria*, *Micrococcus*, and *Streptomyces*, have shown their phosphate-solubilizing properties [19-23]. The potassium-solubilizing activity of the actinobacteria *Streptomyces* and *Nocardiopsis* has been documented [24, 25]. Currently, there are a few reports on zinc solubilization by actinobacteria in the rhizosphere. *Streptomyces nanhaiensis* strain YM4 isolated from the rhizosphere of the rajagra plant exhibited the capacity to solubilize zinc oxide at 41.16 ppm in liquid medium [26]. Verma *et al*. [21] reported that two strains (IARI-HHS1-1 and IARI-HHS1-25) of the genus *Arthrobacter* were obtained from the rhizosphere of wheat and showed halo zone formation around their colonies on solid media containing insoluble zinc compounds. In addition, there have been no reports on isolating zinc-solubilizing actinobacteria from the rhizosphere of tuber crops and legumes.

Soybean (*Glycine max* (L.) Merrill), which is a globally important grain crop, shows zinc deficiency symptoms such as stunted growth, delayed maturity, and interveinal chlorosis of the leaves in sandy soils and where there are high soil pH (calcareous soils) and high soil phosphate levels. [27]. This deficiency has been solved by applying inorganic zinc fertilizers in several forms, including ZnSO_4_, zinc oxide (ZnO), and zinc carbonate (ZnCO_3_) [28]. However, the increasing use of chemical fertilizers has resulted in different adverse types of environmental and human health effects [29]. It is possible to enhance the level of available zinc in the rhizosphere by using zinc-solubilizing actinobacteria as bioinoculants or biofertilizers to increase the growth, yield, and quality of plants. In our study, we used the green vegetable soybean or edamame as a model plant. Vegetable soybean is widely consumed in Asia and America due to its high nutraceutical value and it is an economically important crop [30]. Therefore, our aim in this study was to isolate, screen, and identify zinc-solubilizing actinobacteria from the rhizosphere soil of tuber crops and legumes. The selected strains were also screened for their plant growth-promoting activities (phosphate solubilization, production of IAA, ammonia, and siderophore) and their effects on vegetable soybean seedling growth under greenhouse conditions.

## Materials and Methods

### Sampling Sites and Soil Sample Collection

Twenty-four rhizospheric soil samples were obtained from cassava, sweet potato, and groundnut fields in 4 provinces, Chonburi, Rayong, Kanchanaburi, and Nakorn Sawan of Thailand, as shown in Table 1. The samples were excavated from around the rhizosphere of plants at a depth of 10-15 cm and placed in sterilized plastic bags. The collected soil samples were then air-dried at 30 ± 2°C for 7 days and ground aseptically into fine powder using a mortar prior to use for isolation.

### Isolation of Rhizospheric Actinobacteria

Isolation of actinomycetes was performed by the serial dilution method and spread plate technique. Each dried soil sample (10 g) was suspended in 90 ml of sterile distilled water and subsequently agitated at room temperature on an orbital shaker at 200 ×*g* for 30 min. Then, serial dilution was carried out. Aliquots (0.1 ml) of each dilution were spread on humic acid vitamin agar and starch casein agar supplemented with 50 μg/ml cycloheximide, 25 μg/ml nalidixic acid and 50 μg/ml nystatin to inhibit bacterial and fungal contamination. The plates were incubated at 30± 2°C for up to 14 days and observed for the typical colonies of actinomycetes. The actinomycete colonies were folded and slow growing, chalky, of leathery appearance with an earthy smell, and had aerial and substrate mycelia of different colors [31]. Then, the colonies were picked and purified on yeast extract-malt extract agar medium (ISP-2). The isolated pure cultures were maintained either in slant culture on ISP-2 agar and stored at 4°C or preserved in 20% (v/v) glycerol at -80°C.

### Morphological Characterization

The isolates were grown on ISP2 agar plates for 7-14 days at 30°C. The colony morphology was determined from the color of aerial mycelia, substrate mycelia, and soluble pigment production based on the Inter-Society Colour Council National Bureau of Standards (ISCC-NBS) color name chart [32]. The morphology of the spore-bearing hyphae and spore chains was observed under a light microscope (model CX 31; Olympus, Japan) with a 50× long working distance objective lens (model SLMPLN50X; Olympus).

### Qualitative Zn Solubilization Assay

The ability of the actinobacteria to solubilize zinc was evaluated on Bunt and Rovira agar medium supplemented with 0.1% (w/v) of insoluble zinc salts of ZnO and ZnCO_3_ [33]. Each isolate was separately spotted on the center of an agar plate and incubated at 30°C for 7 days. Then, the colonies exhibiting a solubilization zone were selected and the diameters of the solubilization halo zone and colony were recorded in millimeters (mm). The zinc solubilization index (SI) was calculated according to Sharma *et al*. [34] following the formula:

SI = (colony diameter + halo zone diameter)/(colony diameter).

All experiments were performed at least three times and were also repeated at least three times. The potential of the zinc-solubilizing strains was selected for further quantitative assessment of zinc solubilization.

### Quantitative Zn Solubilization Assay

The selected strains were cultured on ISP-2 agar plates for 7 days. Then, the spores were harvested by flooding the agar plate with sterile 0.1% (v/v) Tween 80 solution and subsequently the obtained suspension was diluted to 1.50 × 10^8^ spores/ml using a hemocytometer. Later, 1 ml of the spore suspension was inoculated into each flask containing 100 ml of Bunt and Rovira broth supplemented with 0.1% ZnO and incubated at 30°C for 7 days with continuous shaking at 200 ×*g* . The uninoculated broth served as a control. After incubation, the culture broth was centrifuged at 8,000 ×*g* for 10 min. For zinc analysis, the culture broth was filtered and 98 ml of filtrate was digested using 2 ml of 6 N HCl. Then, 1 ml of each supernatant was analyzed using an atomic absorption spectrophotometer (Agilent Technologies 200 Series AA, USA) and the total soluble zinc content was expressed in mg/ml. The pH levels of the inoculated and uninoculated supernatants were determined at 0 and 10 days of incubation using a pH meter (Sartorius DOCU-pH+/p11, Germany).

### Evaluation of Plant Growth-Promoting Characteristics of Potent Zinc-Solubilizing Actinobacteria

**Indole-3-acetic acid production.** The evaluation of IAA production was performed based on the colorimetric assay described by Gordon and Weber [35] with some modification. A sample (1 ml) of the spore suspension (1.50 × 10^8^ spores/ml) of each strain was inoculated in 100 ml of ISP-2 broth supplemented with 0.2% (w/v) tryptophan in a shaker (200 ×*g*) at 30 ± 2°C for 7 days. After incubation, the cultures were centrifuged at 8,000 ×*g* for 10 min. For qualitative analysis, the supernatant (250 μl) was dropped on a white ceramic plate, mixed with 250 μl of Salkowski’s reagent and stored in the dark at room temperature for 30 min. Strains producing IAA were recognized by the changing of the plate sample color from yellow to pink. For quantitative determination, 1 ml of the supernatant was mixed with 2 ml of Salkowski’s reagent in a test tube and incubated in the dark for 30 min for color development. The quantity of IAA was measured using a UV‒Vis spectrophotometer (Merck Spectroquant Pharo 300, Germany) at a wavelength of 530 nm. The IAA concentration values were calculated using a standard curve of IAA.

**Phosphate solubilization.** Qualitative estimation of phosphate solubilization was carried out on Pikovskaya agar [36] using tricalcium phosphate (Ca_3_(PO_4_)_2_) as a source of the insoluble form of phosphate. Each of the potent strains was placed on a medium plate using a point inoculation technique. The plates were then incubated at 30°C for 7 days and colonies with clear halo zones were recorded as positive. Subsequently, quantitative estimation of solubilized phosphate by the positive strains was performed using the phosphomolybdate blue complex colorimetric method [37]. First, the selected strains were inoculated with 1 ml of spore suspension (1.50 × 10^8^ spores/ml), and the positive strains were inoculated in a flask of 100 ml of Pikovskaya liquid medium containing 0.5% (w/v) Ca_3_(PO_4_)_2_. The flasks were incubated at 30 ± 2°C for 7 days on a shaker at 200 rpm. After incubation, the culture broth was centrifuged at 8000 ×*g* for 10 min. Then, 200 μl of the supernatant was separately mixed with 1 ml of ammonium molybdate reagent before adding 200 μl of working solution of chlorostatic acid along with shaking. The total volume of the reaction mixture was brought to 5 ml with distilled water. The absorbance of the mixture was measured at 660 nm using a UV‒Vis spectrophotometer (Merck Spectroquant Pharo 300, Germany). The amount of soluble phosphate was determined from the standard curve derived from a regression equation of potassium dihydrogen phosphate (KH_2_PO_4_).

**Siderophore-producing activity**. The potent zinc-solubilizing strains were checked for siderophore-producing activity on universal Chrome azurol S (CAS) agar medium for qualitative assays as described by Schwyn and Neilands [38]. The strains were point-inoculated on CAS agar plates and incubated at 30°C for 7 days. The formation of a yellow‒orange halo zone surrounding the colonies was recorded as a positive result for siderophore production.

**Ammonia production**. The efficient zinc-solubilizing strains were qualitatively and quantitatively tested for ammonia production in peptone water according to Cappucino and Sherman [39]. A sample (1 ml) of spore suspension (1.50 × 10^8^ spores/ml) of the actinobacterial strain was inoculated in 100 ml of peptone water broth and incubated at 30°C with shaking at 200 rpm for 7 days. Uninoculated medium was used as a negative control. In the qualitative test, the supernatant sample (600 μl) was dropped onto a white ceramic plate and 30 μl of Nessler’s reagent was added. The presence of a yellow-to-brown color indicated the production of ammonia and hence was recorded as a positive result. For the quantitative analysis, 4 ml of the appropriate dilution of supernatant was mixed with 200 μl of Nessler’s reagent, and the assay system was kept for 5 min until a yellow color developed. The absorbance was measured at 450 nm using a UV‒Vis spectrophotometer (Merck Spectroquant Pharo 300). The concentration of ammonia was calculated based on a standard curve with the regression equation of ammonium sulfate ((NH_4_)_2_SO_4_).

### Characterization of Selected Strains

**Morphological, Cultural, Physiological and Biochemical Characterization.** Spore chain morphology was studied under a light microscope with a 50× long working distance objective lens following growth on ISP-2 agar at 30 ± 2°C for 7-14 days. Gram staining was also performed. The cultural characteristics were determined on various International *Streptomyces* Project (ISP) media, namely yeast extract-malt extract agar (ISP-2), oatmeal agar (ISP-3), inorganic salt-starch agar (ISP-4), glycerol-asparagine agar (ISP-5), peptone-yeast extract iron agar (ISP-6), and tyrosine agar (ISP-7), at 30 ± 2°C for 7-14 days [40]. The colors of the aerial mycelium, substrate mycelium, spore mass and soluble pigment were observed by comparison with the ISCC-NBS color charts. The production of melanoid pigment was also observed in ISP-6 and ISP-7. Physiological characteristics were determined by different pH levels (3, 4, 5, 6, 7, 8, 9, 10, 11, and 12), sodium chloride concentrations (0.5, 1, 2, 3, 4, and 5% w/v) and temperatures (10, 15, 20, 25, 30, 35, 40, and 45°C) on ISP-2 medium, which was incubated at 30 ± 2°C for 7-14 days [41]. Biochemical tests were performed for nitrate reduction, citrate utilization, triple iron agar test, methyl red (MR) test, Voges-Proskauer (VP) test, hydrogen sulfide (H2S) production, urease, catalase, oxidase, starch hydrolysis, casein hydrolysis, lipid hydrolysis, and gelatin hydrolysis [42, 43]. The utilization of the carbon source was tested on basal medium (ISP-9) supplemented with 1% (w/v) of different sugars, such as D-glucose (positive control), L-arabinose, sucrose, D-xylose, *myo*-inositol, D-mannitol, D-fructose, rhamnose, raffinose, cellulose, and no carbon source (negative control) [40]. Identification of the isomers of diaminopimelic acid in the cell wall hydrolysates were performed by thin-layer chromatography (TLC) [44, 45].

**16S rDNA Sequence Analysis**. For genomic DNA extraction, the selected strains of zinc-solubilizing actinobacteria were cultured in ISP-2 broth on a rotary shaker (200 rpm at 30 ± 2°C) for 7-10 days. After incubation, the cells were harvested using centrifugation at 10,000 ×*g* for 10 min and washed with TE buffer (10 mM Tris/HCl and 1 mM EDTA, pH 8.0). Genomic DNA isolation of the selected strains was extracted using a Genomic DNA Mini Kit (blood/culture cell; Geneaid Biotech Ltd., Taiwan) according to the manufacturer’s instructions. Amplification of the 16S rDNA of each strain was performed using the universal primers 20F (5′-GAGTTTGATCCTGGCTCAG-3′) as the forward primer and 1500R (5′-GTTACCTTGTTACGACTT-3′) as the reverse primer. PCR amplification was carried out using a DNA Engine Dyad Thermal Cycler (Bio-Rad Laboratories, USA). Each 100-μl reaction mixture was composed of template DNA (15-20 ng), 0.2 μM of each primer, 2.5 units of Taq polymerase, 2.0 mM MgCl_2_, 0.2 mM dNTP, and 10 μl of 10 × Taq buffer (750 mM Tris-HCl, 200 mM (NH_4_)_2_SO_4_ and 0.1% Tween 20; pH 8.8). The reaction conditions consisted of an initial denaturation step at 94°C for 3 min, 25 cycles of a denaturation step at 94°C for 1 min, annealing at 50°C for 1 min, elongation at 72°C for 2 min and an amplification step at 72°C for 3 min. The amplification product was checked using gel electrophoresis on a 0.8% (w/v) agarose gel [46-48]. The PCR product was purified using a GenepHlow Gel/PCR Kit (Geneaid Biotech Ltd.). Sequencing of the 16S rRNA gene was performed on an ABI Prism 3730xl DNA analyzer (Applied Biosystems, USA) by Macrogen, Inc. (Korea) using universal primers 27F (5’-AGAGTTTGA TCMTGGCTCAG-3’), 518F (5’-CCAGCAGCCGCGGTAATACG-3’), 800R (5’-TACCAGGGTATCTAATCC-3’) and 1492R (5’ TACGGYTACCTTGTTACGACTT-3’). In the sequence analysis, the 16S rDNA sequences of all primers were assembled using the Cap contig assembly program, an accessory application in the BioEdit (Biological sequence alignment editor) program [49]. The 16S rDNA gene sequences were compared with the related species from the nucleotide database (EzBiocloud server) using the Basic Local Alignment Search Tool (BLAST) program [50]. Multiple sequence alignments were carried out using the Clustal W program in BioEdit Sequence Alignment Editor (version 7.0.0) software [51, 52]. Phylogenetic trees were constructed based on the neighbor-joining algorithm in the MEGA version X software package [53, 54]. The topology of the tree was evaluated using bootstrap analysis with 1,000 replications [55].

### Inoculum Preparation for Pot Experiment

The two potent zinc-solubilizing actinobacteria strains (CME34 and EX51) were cultured on ISP-2 agar plates for 7 days. The spores were scraped off the surface of the culture plate and sterilized with 0.1% (w/v) Tween 80. The spore concentration was adjusted to 1.50 × 10^8^ spores/ml for each strain using a hemocytometer and the resulting suspensions were used as the inoculum in the pot experiment.

### Greenhouse Pot Experiment

The effects of the potent zinc-solubilizing actinobacteria on soybean seedling growth were investigated in a greenhouse of the Central Laboratory and Greenhouse Complex, Faculty of Agriculture at Kamphaeng Saen, Kasetsart University, Kamphaeng Saen Campus, Thailand. Before the experiment, some soil chemical properties—pH, soil organic matter, available phosphorus and exchangeable cations (potassium ions (K^+^), zinc ions (Zn^2+^), and magnesium (Mg^2+^)—were analyzed by the Soil Plant and Agricultural Material Testing and Research Unit, Central Laboratory and Greenhouse Complex, Faculty of Agriculture at Kamphaeng Saen, Kasetsart University, Kamphaeng Saen Campus. The soil had a pH value of 7.65 and contained 16.98% organic matter, 1.21 g/kg available phosphorus, 4.73 g/kg exchangeable K^+^, 12.53 mg/kg exchangeable Zn^2+^, and 0.52 g/kg exchangeable Mg^2+^.

Seeds of green vegetable soybean MJ101 were provided by the Tropical Vegetable Research Center, Kasetsart University Kamphaeng Saen campus. The soybean seeds were surface sterilized with 10% (w/v) sodium hypochlorite (NaClO) for 5 min and washed three times with sterile distilled water. The sterilized seeds were germinated on coco peat for 14 days. Then, they were transferred to plastic pots with a 12-cm diameter containing unsterile soil inside the greenhouse at day/night temperatures of 30°C/25°C, under natural daylight and watered with tap water once a day. The four treatments included: (1) a negative control without inoculation, (2) a positive control (soil containing 0.5 mg/kg of soil ZnSO_4_), (3) inoculation with strain CME34 and (4) inoculation with strain EX51. The inoculation was performed as follows: 5 ml of spore suspensions (1.50 × 10^8^ spores/ml) was applied to the rhizosphere of the seedlings using a sterile syringe at 14 and 30 days after sowing. The control treatments were applied with sterile distilled water instead of the spore suspension. The height of the plants was recorded at intervals of 15 days after planting. After 70 days, the soybean plants were harvested and measured for root length, shoot length, number of pods and number of seeds. The plant dry weight was recorded after drying at 70°C to constant weight. The experiment comprised five replications for each treatment. All experiments were performed in triplicate.

### Statistical Analysis

The results for each experiment are presented as the mean ± SD of three replicates. The experimental data were analyzed using multivariate analysis of variance followed by Tukey’s multiple comparison tests (*p* < 0.05).

## Results

### Isolation of Rhizospheric Actinomycetes

In total, 200 actinomycete isolates were obtained from 24 rhizosphere soil samples (93 isolates from casava rhizospheres, 66 isolates from sweet potato rhizospheres, and 41 isolates from groundnut rhizospheres, Table 2). The classification based on the appearance of colonies and the morphology of aerial mycelium and spores revealed that the isolates comprised ten genera: *Streptomyces*, *Microbispora*, *Micromonospora*, *Streptosporangium*, *Dactylosporangium*, *Actinomadura*, *Nocardia*, *Amycolatopsis*, *Pseudonocardia*, and *Nonomuraea*. The predominant genera were *Streptomyces* (n = 107, 53.5%), followed by *Micromonospora* (n = 24, 12%), *Actinomadura* (n = 18, 9%), *Microbispora* (n = 17, 8.5%), *Nocardia* (n = 14, 7%), *Streptosporangium* (n = 5, 2.5%), *Amycolatopsis* (n = 5, 2.5%), *Dactylosporangium* (n = 4, 2%), *Pseudonocardia* (n = 4, 2%), and *Nonomuraea* (n =1, 2%). The typical morphological structure of each genus is shown in Fig. S1.

### Qualitative Assessment of Zinc Solubilization

The zinc-solubilizing ability of each strain which was represented by the diameter of the halo zones and the zinc SI is shown in Table 3. Out of 200 isolates, 16 isolates showed clear halo zones around colonies and thus indicated the ability to solubilize zinc compounds. All 16 isolates showed halo zones on the ZnO-supplemented medium, while only 14 isolates showed zinc solubilization zones on the ZnCO_3_ medium. Two strains (EX51 and CME34) showed maximum zinc solubilization on ZnO-supplemented medium with halo zones of 26.29 and 22.33 mm, respectively. On ZnCO_3_-supplemented medium, EX51 showed similar solubilization with averaged halo zones of 19.36 mm, followed by CME34 with a 16.23-mm halo zone. Four strains had SI index values greater than 3.0, with the highest value being for EX51 (3.78 ± 0.18), followed by CME34 (3.36 ± 0.08), RME15 (3.18 ± 0.11) and AH32 (3.11 ± 0.17), all on the ZnO-supplemented agar medium.

### Quantitative Assessment of Zinc Solubilization

The four selected strains, EX51, CME34, RME15 and AH32, with SI index values exceeding 3.00, were further analyzed for their zinc solubilization in liquid medium for 7 days (Table 4). The incubation time was determined during the exponential phase of growth. The four strains showed a similar trend to the growth profile in Bunt and Rovira broth with 0.1% ZnO (w/v) (Fig. S2). The results revealed that strain CME34 had the highest amount of soluble zinc (529.71 μg/ml), followed by EX51 (243.58 μg/ml), RME15 (46.46 μg/ml), and AH32 (35.62 μg/ml) in the culture filtrate. The maximum pH reduction was observed in strain CME34 (5.24 ± 0.03), followed by EX51 (5.55 ± 0.10), RME15 (5.69 ± 0.22), and AH32 (6.51 ± 0.03). Apparently, CME34 and EX51 strains exhibited the strongest zinc solubilization ability and were selected for further studies.

### Plant Growth-Promoting Characteristics of the Selected Zinc-Solubilizing Strains

Qualitative analysis of the culture filtrate revealed that the strains CME34 and EX51 had positive results for IAA and ammonia production and phosphate solubilization (Table 5). Further quantification revealed that the concentrations of ammonia produced by the CME34 and EX51 strains were 303.61 ± 5.01 and 361.09 ± 5.04 μg/ml, respectively. Likewise, the strains CME34 and EX51 were able to synthesize IAA in liquid medium with production levels of 4.59 ± 0.06 and 4.13 ± 0.10 μg/ml, respectively. For phosphate solubilization, a high content of soluble phosphorus was produced by EX51(114.00 ± 5.29 μg/ml), followed by CME34 (61.67 ± 4.16 μg/ml) after 7 days of incubation.

### Identification of Selected Zinc-Solubilizing Strains

Morphologically, the colonies on ISP-2 agar of the strains CME34 and EX51 showed light-yellow substrate mycelia, white aerial mycelium, and light-gray spore mass. Both strains were observed to be gram-positive, and spores were arranged in straight chains. The cultural characteristics of strains CME34 and EX51 on different types of ISP media showed good growth on all media. Melanin and other soluble pigments of these strains were not produced. The colors of the substrate mycelium and aerial mycelium of the two strains are summarized in Table 6. The physiological and biochemical characteristics are shown in Table 7. Strain CME34 was able to grow at temperatures between 25 and 30°C (optimum at 30°C) and pH value ranging from 6.0 to 11.0 (optimum at pH 7). Strain EX51 was able to grow at temperatures between 15 and 30°C (optimum at 30°C) and pH value ranging from 6.0 to 10.0 (optimum at pH 7). Strain CME34 tolerated sodium chloride up to 2% (w/v), whereas strain EX51 tolerated sodium chloride up to 1% (w/v). Both strains could use D-glucose, L-arabinose, sucrose, D-xylose, *myo*-inositol, D-mannitol, D-fructose, rhamnose, raffinose, and cellulose as the carbon source. These strains were also able to hydrolyze casein, lipid, starch, and gelatin but did not show urea hydrolysis. The two strains were positive for catalase and indole production but negative for oxidase, citrate utilization, MR test, VP test, and H2S production. Strain CME34 was positive for nitrate reduction, while strain EX51was negative. All selected strains showed the presence of *LL*-diaminopimelic acid (*LL*-DAP) in whole-cell hydrolysates.

**Fig. 1:**
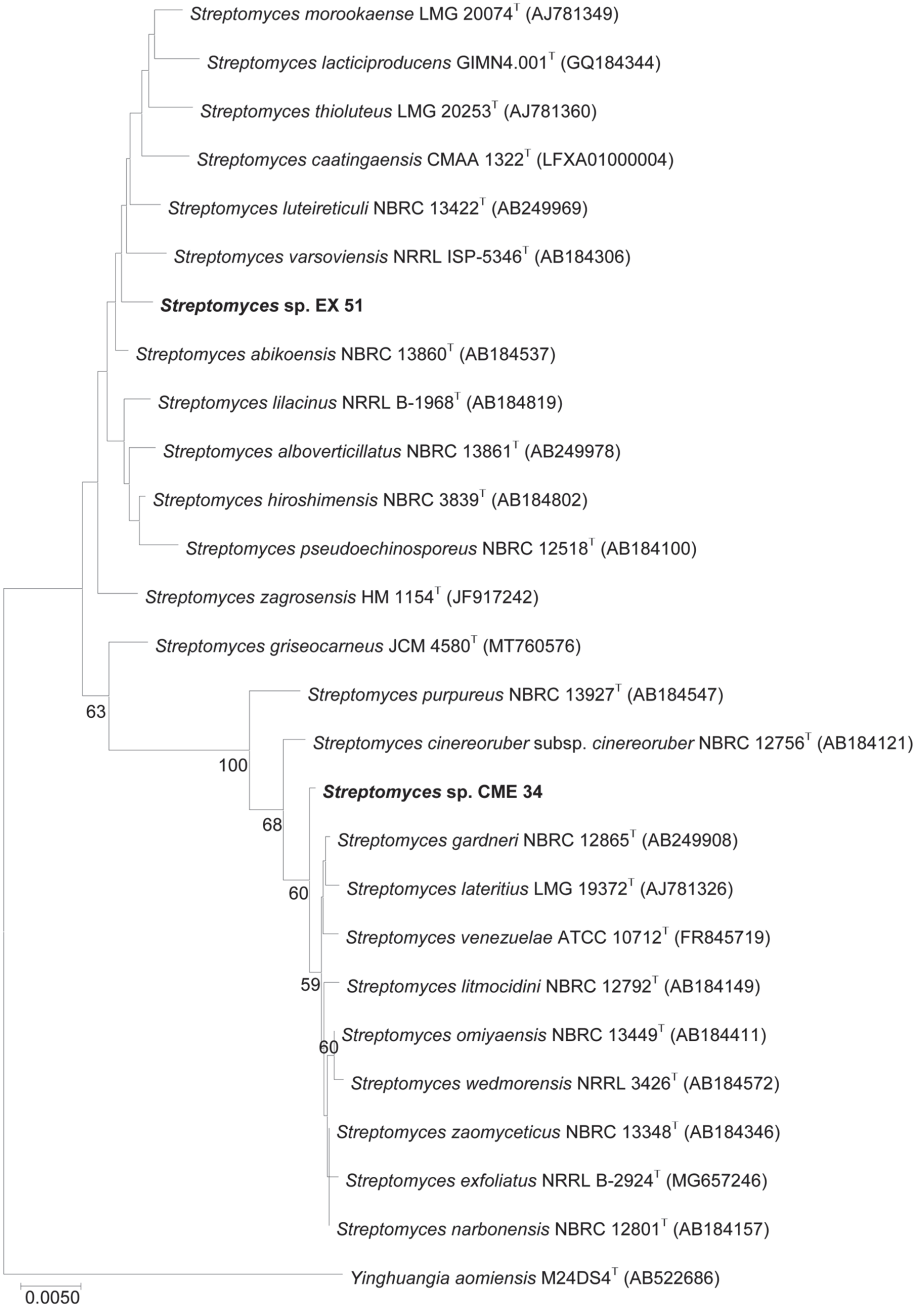
Phylogenetic tree showing relationships between two zinc-solubilizing strains and related strains of *Streptomyces* sp. based on the 16S rRNA gene sequences. The optimal tree had a branch length sum of 0.11. Percentages at the nodes represent the levels of the bootstrap support from 1,000 resampled datasets; only values greater than 50% are indicated. The scale bar indicates 0.005 substitutions per nucleotide position.

The almost complete 16S rRNA gene sequences of the strain CME34 (1,419 bp) showed the highest similarity (99.79%) with *Streptomyces omiyaensis* NBRC 13449 (AB184411) and *Streptomyces zaomyceticus* NBRC 13348 (AB184346). Isolate EX51 (1,416 bp) demonstrated 99.50% similarity to *Streptomyces abikoensis* NBRC 13860 (AB184537). The phylogenetic relationships of these strains are shown in Fig. 1. The phylogenetic tree of the 16S rRNA gene showed that strain CME34 was clustered with *Streptomyces gardneri* NBRC 1865^T^, *Streptomyces lateritius* LMG 19372^T^, *Streptomyces venezuelae* ATCC 10712^T^, *Streptomyces litmocidini* NBRC 12792^T^, *S. omiyaensis* NBRC 13449^T^, *Streptomyces wedmorensis* NRRL 3426^T^, *S. zaomyceticus* NBRC 13348^T^, *Streptomyces exfoliatus* NRRL B-2924, *Streptomyces narbonensis* NBRC 12801^T^, and *Streptomyces cinereoruber* subsp. *cinereoruber* NBRC 12756^T^. Strain EX51 was closer to *S. abikoensis* NBRC 13860 than to the other strains.

### Greenhouse Pot Experiments

**Fig. 2:**
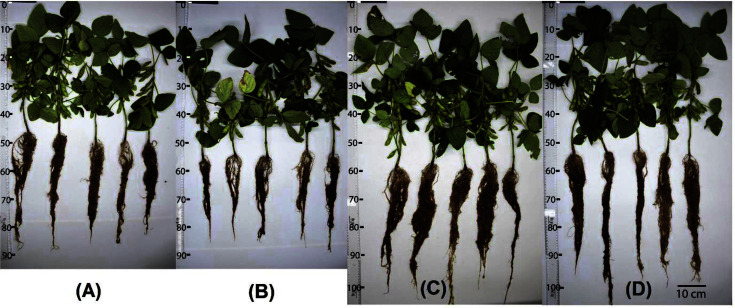
Effects of zinc-solubilizing *Streptomyces* on the growth of vegetable soybeans at 70 days after sowing: (**A**) uninoculated control; (**B**) positive control (soil amended with 0.5 mg ZnSO_4_/kg soil); (**C**) inoculated with CME34 strain; (**D**) inoculated with EX51 strain.

The selected zinc-solubilizing strains were treated for growth promotion of vegetable soybean seedling under greenhouse conditions. At 70 days after sowing (Table 8, Fig. 2), the two strains significantly enhanced several vegetable soybean seedling growth characteristics, namely, shoot length, root length, plant dry weight, number of pods and number of grains compared to the uninoculated control and the positive control. The inoculated treatment of strain CME34 had the highest shoot length (33.57 ± 3.40 cm) and the maximum root length (54.03 ± 3.52 cm) compared to the control group. Furthermore, the inoculation of the CME34 strain showed the most significant ability to increase the shoot length (27.98%), root length (24.09%), plant dry weight (45.34%), number of pods per plant (153.97%) and number of seeds per plant (121.01%) compared to the uninoculated control. Strain EX 51 also significantly increased shoot length (19.98%), root length (15.96%), plant dry weight (30.16%), number of pods per plant (129.87%) and number of seeds per plant (80.95%) compared to the control without inoculation.

## Discussion

In the present study, 200 actinomycete isolates were obtained from sweet potato, cassava, and groundnut rhizospheric soil. Based on the preliminary morphological and physiological characteristics, the isolates were classified into 10 genera. The results supported that the rhizosphere provides various nutrient sources released from root exudates that are beneficial for the activity and diversity of rhizosphere microorganisms [56]. The predominance of the genus *Streptomyces* (53.5%) we observed was consistent with other studies that also examined the rhizosphere of different plants [57, 58].

The screening for the solubilization of inorganic zinc showed that 16 isolates (8%) possessed the ability to solubilize inorganic zinc based on their production of halo zones around their colonies. Furthermore, we observed that almost all the zinc-solubilizing actinobacteria more effectively solubilized ZnO than ZnCO_3_. These findings were in accordance with other reports showing that zinc-solubilizing rhizobacteria had higher solubilizing ability in the medium containing ZnO [26, 59, 60]. Among the strains possessing the highest SI values in agar medium (AH32, CME34, RME15 and EX51), strains CME34 and EX51 had the highest soluble zinc production of 529.71 and 243.58 μg/ml, respectively, in culture broth after 7 days of incubation (Table 4). Both strains belong to the *Streptomyces* genus. Our results correlated well with other studies; for example, *S. narbonensis* strain 68 showed good ability with a zinc-solubilizing content of 272.2 μg/ml in solubilization assay medium containing ZnO [61]. *S. nanhaiensis* strain YM4 had a zinc solubilization of 41.66 μg/ml in minimal medium supplemented with 0.1% (w/v) ZnO [26]. In our study, the pH reduction in the culture medium was correlated with increasing soluble zinc concentration. The maximum reduction in pH and increased zinc solubilization by the CME34 strain (pH 5.24) were observed in the culture broth after incubation. Acidification of the culture broth resulting from the secretion of various organic acids from zinc-solubilizing strains has been suggested as a major mechanism responsible for zinc solubilization [62]. Several studies have suggested that gluconic acid and its derivatives (2-ketogluconic acid and 5-ketogluconic acid) produced in culture medium play an important role in the solubilization of insoluble forms of zinc with a decrease in pH [13, 63, 64].

The strains CME34 and EX51 had IAA production of 4.59 and 4.13 μg/ml, respectively. These results have also been reported to produce IAA by zinc-solubilizing rhizobacteria [65, 66]. In addition, *Streptomyces* strains from the rhizospheres of different plants can synthesize IAA as a plant growth substance [22, 67]. Here, the selected strains showed a high amount of ammonia in peptone water medium (Table 5). Ammonia production by rhizobacteria increased the accumulation or supplementation of nitrogen in the surrounding soil, which may have supplemented the availability of nitrogen to the host plants [68]. For phosphate solubilization, the two strains CME34 and EX51 showed capacities to solubilize phosphate, with available phosphate contents of 61.67 and 114.00 μg/ml on Pikovskaya’s liquid medium, respectively. Most *Streptomyces* strains isolated from rhizospheres have been widely reported for their ability to solubilize insoluble phosphate [69, 70]. Hence, two selected strains were identified by morphological, physiological, and biochemical characteristics and 16S rRNA gene sequencing. These strains belonged to the genus *Streptomyces*. Strain CME34 was 99.79% similar to *Streptomyces omiyeansis* NRBC 13449 and *S. zaomyceticus* NBRC 13348, and strain EX51 showed high similarity (99.50%) to *S. abikoensis* NBRC 13860.

To our knowledge there have been no reports on plant growth promotion by zinc-solubilizing *Streptomyces* strains. The present study revealed that the growth of vegetable soybean seedling inoculated with zinc-solubilizing *Streptomyces* (CME34 and EX51) was superior to that of the uninoculated control and the positive control treatment (Table 8). In addition, the inoculation of vegetable soybean seedling with *Streptomyces* sp. CME34 increased the shoot length, root length, number of pods, number of seeds and plant dry weight by 27.98, 24.09, 45.34, 153.97 and 121.01%, respectively, over the uninoculated control. Our results are consistent with other reports on zinc solubilization to improve plant growth by other bacterial genera [15, 71]. The results of this pot experiment were well supported by the results of zinc solubilization assay, whereby *Streptomyces* was capable of producing organic acids to solubilize insoluble forms of zinc in soil and thus enhanced zinc availability for the plants. In addition, the *Streptomyces* in the present study solubilized phosphate, produced IAA and released NH3, which were all beneficial for plant growth.

In conclusion, we isolated and screened for the potential of zinc-solubilizing rhizospheric actinomycetes. The selected strains (CME34 and EX51), belonging to the genus *Streptomyces*, showed a high ability of zinc solubilization and exhibited various plant growth-promoting activities. The inoculation of CME34 or EX51 on vegetable soybean plants significantly increased shoot length, root length and biomass compared to the uninoculated control. Our results suggested that either of the two *Streptomyces* strains could be used as a bioinoculant to increase the available zinc in soils and improve the growth and yield of plants. Further studies are required to evaluate the effect of co-inoculation in both strains and the combination effect of zinc-solubilizing *Streptomyces* and zinc fertilizer for promoting growth and yield of vegetable soybean or other crops in greenhouse experiments and field trials. In addition, a correlation was investigated between the populations of zinc-solubilizing *Streptomyces* and the level of soil zinc availability and plant growth-promoting activities in the rhizosphere soil after both short and long terms of cropping.

## Supplemental Materials

Supplementary data for this paper are available on-line only at http://jmb.or.kr.

## Figures and Tables

**Table 1:** Location of sweet potato, cassava and peanut rhizosphere soil used for isolation of rhizospheric actinobacteria.

Sampling sites	Geographical coordinates	Plant rhizosphere soil	Number of samples
Ban Khai district, Rayong province	12° 44′ 11.58′′ N, 101° 18′ 53.27′′ E 12° 44 12.09′′ N, 101° 18′ 53.25′′ E 12° 44 42.13′′ N, 101° 20′ 52.42′′ E 12° 44 10.99′′ N, 101° 18′ 52.05′′ E	Sweet potato Sweet potato Cassava Groundnut	3 3 2 3
Sri Racha district, Chonburi province	13° 07′ 25.40′′ N, 100° 55′ 46.00′′ E 13° 10′ 11.40′′ N, 100° 56′ 20.80′′ E	Cassava Cassava	2 2
Phanom Thuan district, Kanchanaburi province	14° 11′ 31.90′′ N, 99° 34′ 31.10′′ E	Cassava	6
Phaisali district, Nakhon Sawan province	13° 23′ 24.00′′ N, 100° 36′ 53.20′′ E	Groundnut	3

**Table 2:** Occurrence and distribution of actinobacteria in different rhizospheric soil samples.

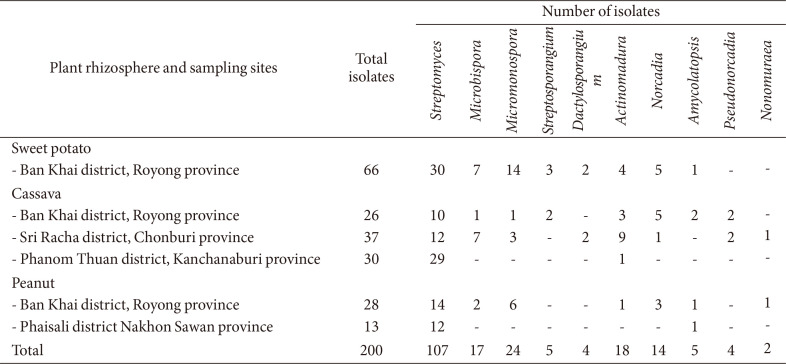

**Table 3:** Colony diameter, halo zone diameter and zinc solubilization index of the 16 actinobacterial isolates on Bunt and Rovira supplemented with 0.1% ZnO and ZnCO_3_.

Strain code	Colony diameter (mm)		Halo zone diameter (mm)		Solubilization index (SI)	

	ZnO	ZnCO_3_	ZnO	ZnCO_3_	ZnO	ZnCO_3_
AH 23	7.30 ± 1.14 ^bdf^	6.27 ± 0.21 ^bde^	9.45 ± 0.95 ^deg^	8.22 ± 0.82 ^def^	2.30 ± 0.08 ^bef^	2.31 ± 0.10 ^bde^
AH 32	6.77 ± 0.15^d^	6.00 ± 0.17^d^	14.33 ± 1.45^c^	10.64 ± 0.84^c^	3.11 ± 0.17^d^	2.77 ± 0.10^c^
AH 34	7.87 ± 1.16 ^ade^	6.83 ± 0.35 ^bcd^	12.77 ± 1.00 ^bcde^	9.98 ± 1.40 ^bde^	2.63 ± 0.11 ^cde^	2.46 ± 0.14 ^bc^
AH 35	8.57 ± 0.59^cd^	7.03 ± 0.21^acd^	11.87 ± 0.59^ade^	10.31 ± 0.86 ^ace^	2.39 ± 0.12 ^bef^	2.46 ± 0.08 ^bc^
CME 34	9.47 ± 0.49^b^	8.23 ± 0.45^b^	22.33 ± 0.61 ^a^	16.23 ± 0.95^b^	3.36 ± 0.08^b^	2.97 ± 0.02^b^
CME 35	7.90 ± 1.05^bde^	8.03 ± 0.21^bc^	12.00 ± 0.80 ^bce^	12.38 ± 0.74^ab^	2.53 ± 0.17 ^cde^	2.54 ± 0.06^ab^
CME 44	9.13 ± 1.53^bc^	7.97 ± 0.47^ac^	15.33 ± 0.94^bcd^	11.51 ± 0.95^ac^	2.70 ± 0.17 ^bdf^	2.44 ± 0.05^bc^
EX 42	6.57 ± 0.49 ^deg^	6.40 ± 0.26^bde^	11.85 ± 1.31 ^ade^	9.60 ± 0.66 ^cdf^	2.80 ± 0.07 ^acd^	2.50 ± 0.05^ab^
EX 46	7.90 ± 0.10^bde^	7.30 ± 0.46 ^ad^	14.55 ± 0.59 ^ab^	10.36 ± 0.86 ^acd^	2.84 ± 0.08^ac^	2.42 ± 0.03 ^bcd^
EX 51	9.50 ± 0.75^a^	9.13 ± 0.32^a^	26.29 ± 1.21^a^	19.36 ± 0.51^a^	3.78 ± 0.18^a^	3.12 ± 0.08^a^
EX 54	6.17 ± 0.15^deg^	6.07 ± 0.06 ^bdf^	8.78 ± 0.87 ^dfg^	8.96 ± 0.99^def^	2.42 ± 0.11 ^cef^	2.43 ± 0.15^bcd^
NAH 13	6.60 ± 0.53^deg^	0.00 ± 0.00	7.83 ± 0.61^dfg^	0.00 ± 0.00	2.19 ± 0.13 ^defg^	0.00 ± 0.00
NAH 22	6.70 ± 0.95^acg^	7.00 ± 0.20 ^acd^	11.81 ± 0.75 ^ade^	10.44 ± 0.73 ^acd^	2.77 ± 0.15 ^bde^	2.49 ± 0.06^ab^
OIB 22	7.77 ± 0.95^bdef^	0.00 ± 0.00	10.91 ± 0.65 ^bce^	0.00 ± 0.00	2.41 ± 0.11 ^cef^	0.00 ± 0.00
OIB34	7.60 ± 0.62^bdfg^	7.60 ± 0.20 ^bc^	13.75 ± 1.59^bc^	9.81 ± 0.35^bde^	2.81 ± 0.16 ^acd^	2.29 ± 0.03^bdf^
RME 15	8.10 ± 0.61^c^	7.87 ± 0.21^c^	17.65 ± 1.08 ^b^	14.22 ± 1.01^c^	3.18 ± 0.11^c^	2.81 ± 0.08^c^

Data presented as means of 3 replicates ± SD., Means sharing the same letter do not differ significantly (*p* < 0.05).

**Table 4:** Quantity of soluble zinc resulting from the solubilization of ZnO by selected rhizospheric actinomycete strains and the pH after incubation.

Strain code	Quantity of soluble zinc (μg/ml)	pH after incubation
AH 32	35.62 ± 2.07^bd^	6.51 ± 0.03^bc^
CME 34	529.71 ± 10.70^a^	5.24 ± 0.03^a^
EX 51	243.58 ± 14.26^b^	5.55 ± 0.10 ^b^
RME 15	46.46 ± 3.02^bc^	5.69 ± 0.22^b^

Data presented as means of 3 replicates ± SD., Means sharing the same letter do not differ significantly (*p* < 0.05).

**Table 5:** Qualitative and quantitative analysis of plant growth-promoting traits of selected zinc-solubilizing strains.

Strain code	Qualitative analysis					Quantitative analysis		

	NH_3_	IAA	SID	PS	KS	NH_3_ (μg/ml)	IAA (μg/ml)	PS (μg/ml)
CME 34	+	+	-	+	-	303.61 ± 5.01	4.59 ± 0.06	61.67 ± 4.16
EX 51	+	+	-	+	-	361.09 ± 5.04	4.13 ± 0.10	114.00 ± 5.29

Data presented as means of 3 replicates ± SD. **NH_3_**: ammonia production; **IAA**: indoleacetic acid production; **SID**: siderophore production; **PS**: phosphate solubilization, **KS**: potassium solubilization; The symbol, + represents the presence of the trait, - represents the absence of the trait.

**Table 6:** Cultural characteristics of strains CME34 and EX51 on different ISP media.

Strain	Medium	Growth	Substrate mycelium	Aerial mycelium	Spore mass	Soluble pigment
CME34	ISP2	Good	Light yellow	White	Light gray	None
	ISP3	Good	Yellowish white	Yellowish gray	Light grayish brown	None
	ISP4	Good	Light yellow	Moderate yellow	Light gray	None
	ISP5	Good	Light yellow	Light yellow	White	None
	ISP6	Good	Light yellow	White	Light gray	None
	ISP7	Good	Light yellow	Light yellow	Light gray	None
EX51	ISP2	Good	Light yellow	White	Light gray	None
	ISP3	Good	Yellowish white	Grayish yellow	Yellowish gray	None
	ISP4	Good	Light yellow	Dark yellow	Yellowish white	None
	ISP5	Good	Light yellow	Brilliant yellow	White	None
	ISP6	Good	Light yellow	Moderate yellow	Light gray	None
	ISP7	Good	Light yellow	Light yellow	White	None

**Table 7:** Morphological, physiological and biochemical characteristics of strains CME34 and EX51.

Characteristic	CME34	EX51
*Morphological characteristics* Spore chain	Straight	Straight
Spore mass	Light gray	Light gray
Aerial mycelium colour	White	White
Substrate mycelium colour	Light yellow	Light yellow
*Physiological characteristics*		
Production of melanoid pigment	-	-
Range of temperature for growth	25°C to 30°C	15°C to 30°C
Optimum temperature	30°C	30°C
Range of pH for growth	6 to 11	6 to 10
Optimum pH	7	7
NaCl tolerance	0.5 to 2% (w/v)	0.5 to 1% (w/v)
*Biochemical characteristics*		
Gram staining	Gram-positive	Gram-positive
Melanin on tyrosine agar	-	-
Triple iron agar	K/A	K/A
Indole test	+	+
MR test	-	-
VP test	-	-
Citrate utilization	-	-
Cell wall amino acids	*LL*-DAP	*LL*-DAP
Starch hydrolysis	+	+
Casein hydrolysis	+	+
Gelatin hydrolysis	+	+
Lipid hydrolysis	+	+
Urea hydrolysis	-	-
Catalase	+	+
Oxidase	-	-
Nitrate reduction	+	-
H_2_S production	-	-
*Utilization of carbon sources*		
D-Glucose	+	+
L-Arabinose	+	+
Sucrose	+	+
D-Xylose	+	+
*myo*-Inositol	-	+
D-Mannitol	+	+
D-Fructose	+	+
Rhamnose	+	+
Raffinose	+	+
Cellulose	+	+

**Table 8:** Effect of individual zinc-solubilizing *Streptomyces* on vegetable soybean seedling growth promotion.

Treatment	Growth parameter				

	Shoot length (cm)	Root length (cm)	Pod number (per plant)	Seed number (per plant)	Plant dry weight (g/plant)
Non-inoculated control	26.23 ± 1.76^b^	43.54 ± 3.81^d^	6.93 ± 1.28^d^	16.80 ± 3.08^d^	5.47 ± 0.31^c^
Positive control, (0.5 mg ZnSO_4_/kg soil)	27.63 ± 2.08^b^	47.65 ± 3.54^c^	10.40 ± 1.18^c^	27.33 ± 3.20^c^	5.71 ± 0.25^c^
Inoculation with CME 34	33.57 ± 3.40^a^	54.03 ± 3.52^a^	17.60 ± 2.26^a^	37.13 ± 2.64^a^	7.95 ± 0.56^a^
Inoculation with EX 51	31.47 ± 2.30^a^	50.49 ± 2.36^b^	15.93 ± 1.71^b^	30.40 ± 2.85^b^	7.12 ± 0.49^b^

Data are mean ± SD of five replications from three independent experiments; Mean values followed by different lowercase superscripts in column of each treatment are significantly different at *p* < 0.05 according to a Tukey test.
