# Supplementary material for: Zinc-Solubilizing Streptomyces spp. as Bioinoculants for Promoting the Growth of Soybean (Glycine max (L.) Merrill)
Source: J Microbiol Biotechnol. 2022 Oct 17;32(11):1435–46. doi: 10.4014/jmb.2206.06058 (PMC9720072; doi:10.4014/jmb.2206.06058)
Supplement: Supplementary file 1 [file jmb-32-11-1435-supple.pdf]

Supplementary Figures

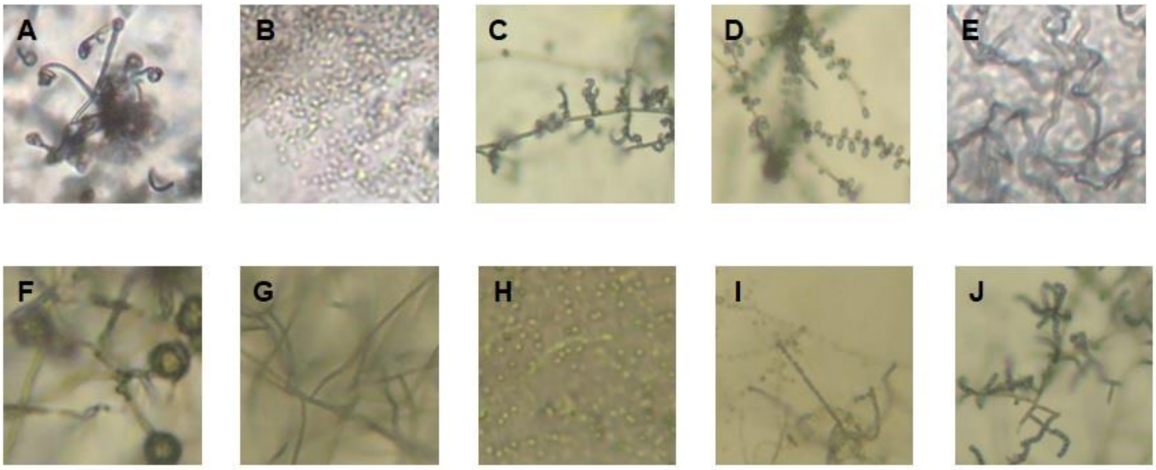

Fig. 1S. Microscopic observation of the actinobacteria in found in different rhizopheric soil samples: (**A**) *Streptomyces*, (**B**) *Micromonospora*, (**C**) *Actinomadura*, (**D**) *Microbispora*, (**E**) *Nocardia*, (**F**) *Streptosporangium*, (**G**) *Amycolatopsis*, (**H**) *Dactylosporangium*, (**I**) *Pseudonocardia*, (**J**) *Nonomuraea*

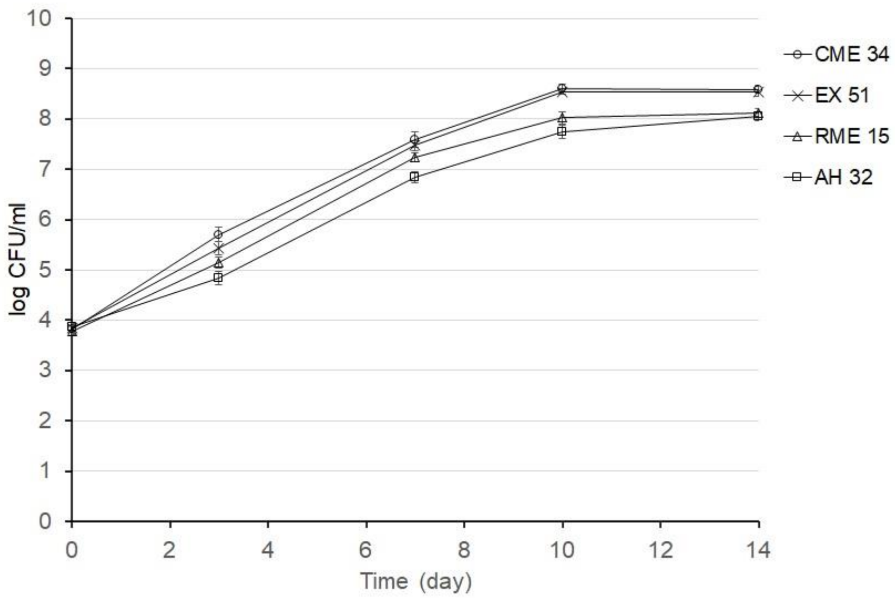

Fig. 2S. Growth curve of strains CME 34, EX 51, RME 15 and AH 32 were grown in Bunt and Rovira broth containing 0.1% ZnO (w/v) at 0, 3, 7, 10 and 14 days after inoculation. Data presented as means of 3 replicates ± SD.
